# Linking Religious Identity, Participation, and Faith to Domains of Mental Health in Late Life

**DOI:** 10.1093/geroni/igab046.1097

**Published:** 2021-12-17

**Authors:** Eva Kahana, Poshan Dahal, Tirth Bhatta, Polina Ermoshkina

**Affiliations:** 1 Case Western Reserve University, Cleveland, Ohio, United States; 2 Case Western Reserve University, Case Western Reserve University, Ohio, United States; 3 University of Nevada, Las Vegas, Nevada, United States

## Abstract

Religiosity in late life has been linked to psychological well-being outcomes. However, there has been insufficient attention to complex associations between different domains of religiosity and domains of psychological wellbeing. We explored associations between religious identity, religious participation, religious coping (trust in God), and mental health indicators of depressive symptoms, life satisfaction, and positive/negative affect among 797 independent, retirement community-dwelling older adults. At baseline, religious identity (expressed as self- concept) and religious participation (church attendance) each were associated with fewer depressive symptoms (b=-0.47, p<0.05; b=-0.19, p<0.05). Religious identity, however, was significantly associated with both life satisfaction and positive affects but not with negative affect. Religious coping was associated with greater life satisfaction and positive affect. Our longitudinal analysis documented a statistically significant decline in depressive symptoms, and increase in life satisfaction and positive affect, with corresponding increase in religious identity over time. However, changes in religious identity did not lead to significant changes in negative affect over time. Religious coping and church attendance fully explained the influence of religious identity on changes in life satisfaction. Although the influence of religious identity on depressive symptoms and positive affect was weakened, its significant influence was maintained even after the consideration of religious coping and church attendance. Beyond religious identity, we also observed a significant increase in positive affect with a corresponding increase in religious coping. Overall, our findings support expectations that religious identification and practices are associated with greater psychological well-being among community dwelling old- old adults.

